# Peg-in-hole assembly skill imitation learning method based on ProMPs under task geometric representation

**DOI:** 10.3389/fnbot.2023.1320251

**Published:** 2023-11-09

**Authors:** Yajing Zang, Pengfei Wang, Fusheng Zha, Wei Guo, Chao Zheng, Lining Sun

**Affiliations:** ^1^School of Mechatronics Engineering, State Key Laboratory of Robotics and System, Harbin Institute of Technology, Harbin, China; ^2^Wuhan Second Ship Design and Research Institute, Wuhan, China

**Keywords:** peg-in-hole assembly, imitation learning, Behavioral Cloning, probabilistic movement primitives, robot manipulation planning

## Abstract

**Introduction:**

Behavioral Cloning (BC) is a common imitation learning method which utilizes neural networks to approximate the demonstration action samples for task manipulation skill learning. However, in the real world, the demonstration trajectories from human are often sparse and imperfect, which makes it challenging to comprehensively learn directly from the demonstration action samples. Therefore, in this paper, we proposes a streamlined imitation learning method under the terse geometric representation to take good advantage of the demonstration data, and then realize the manipulation skill learning of assembly tasks.

**Methods:**

We map the demonstration trajectories into the geometric feature space. Then we align the demonstration trajectories by Dynamic Time Warping (DTW) method to get the unified data sequence so we can segment them into several time stages. The Probability Movement Primitives (ProMPs) of the demonstration trajectories are then extracted, so we can generate a lot of task trajectories to be the global strategy action samples for training the neural networks. Notalby, we regard the current state of the assembly task as the via point of the ProMPs model to get the generated trajectories, while the time point of the via point is calculated according to the probability model of the different time stages. And we get the action of the current state according to the target position of the next time state. Finally, we train the neural network to obtain the global assembly strategy by Behavioral Cloning.

**Results:**

We applied the proposed method to the peg-in-hole assembly task in the simulation environment based on Pybullet + Gym to test its task skill learning performance. And the learned assembly strategy was also executed on a real robotic platform to verify the feasibility of the method further.

**Discussion:**

According to the result of the experiment, the proposed method achieves higher success rates compared to traditional imitation learning methods while exhibiting reasonable generalization capabilities. It shows that the ProMPs under geometric representation can help the BC method make better use of the demonstration trajectory and thus better learn the task skills.

## 1. Introduction

Assembly operations are a critical process in manufacturing, involving the connection and assembly of various components to create products, and they encompass nearly every aspect of the manufacturing industry (Zhao et al., 2023). Industrial robots, serving as the primary execution devices in assembly production, face the crucial challenge of rapidly acquiring assembly operation skills, which significantly impact the development of the manufacturing sector.

In order to equip the robot with manipulation skills effectively, a variety of robot task learning methods have emerged in recent years (Bing et al., 2022a,b). Among these methods, reinforcement learning (Luo et al., 2019; Bing et al., 2023b) and imitation learning (Ehlers et al., 2019; Huang et al., 2019) are the most common methods. Reinforcement learning methods are designed to give robots the ability to learn autonomously, which enables the robot to learn the unknown tasks completely independently through neural network agents. As for the tasks for which humans have sufficient operational experience, imitation learning methods can make use of human operational experience to achieve rapid learning of task skills and are usually more widely used.

Demonstration learning is a common method for robot skill acquisition. This approach extracts operation features from perceptual data obtained during human-demonstrated tasks. It then models operational skills using mathematical techniques and, finally, replicates and generalizes actions based on skill parameters. This methodology mimics the human learning process, enabling robots to effectively inherit assembly experiential knowledge already possessed by humans.

Traditional demonstration learning methods mainly fall into two categories: those based on mathematical models and those based on neural networks. Mathematical model-based methods, such as Dynamic Movement Primitives (DMP) (Chen et al., 2017; Liu et al., 2020), ProMPs (Paraschos et al., 2018), and Task-Parameterized Gaussian Mixture Model (TP-GMM) (Rozo et al., 2015, 2016; Silvério et al., 2019), model demonstration data by establishing trajectory models. These methods learn operational knowledge by adjusting and optimizing the parameters of mathematical models, similar to how humans adjust and refine their modeling process based on relevant experience. While mathematical approaches can provide precise models, they often require a high level of accuracy in input state information. Consequently, it is challenging for these models to generalize knowledge to the entire global task space.

The paper is organized as follows: Section 2 summarizes the existing research related to the proposed method; Section 3 introduces the implementation detail and the quantitative evaluation of the proposed method; Section 4 provides a series of experimental results and analysis. In Section 5, conclusions are drawn and directions for future work are provided.

Even though networks is widely known to be used in machine learning categories (Bing et al., 2023a,c), they are also beginning to be taken good use for imitation learning methods. Till now network-based imitation learning methods involve sampling human-operated skills and then training neural networks using the acquired sample data to achieve skill learning. As of now, neural network-based imitation learning methods primarily include BC methods (Li et al., 2022), where neural networks are directly trained, and Generative Adversarial Imitation Learning (GAIL) methods (Tsurumine and Matsubara, 2022), which approximate strategies through generative adversarial techniques. Neural network-based imitation learning methods excel in strategy approximation. However, due to the typically sparse nature of human demonstration data, training neural networks to obtain global strategies with a limited sample size poses a significant challenge.

Peg and hole assembly, a representative assembly task, finds widespread applications in aerospace manufacturing, shipbuilding, precision instrument manufacturing, and other fields. However, the diverse ways pegs and holes interact result in a multitude of task states and corresponding skill strategies. Consequently, collecting strategy samples becomes exceptionally challenging, making it even more difficult to achieve global skill learning in the task space through neural network-based imitation learning methods.

To address this issue, we propose a method that leverages a compact task representation space to achieve data augmentation. With limited demonstration data, we employ the ProMPs method to establish a mathematical model for global task space strategy. We then use BC to train neural networks, enabling global skill learning in the task space. This paper's contributions are as follows:

1. We extracted human demonstration data and mapped it to a feature space using the method described in Zang et al. (2023).
2. Using the trajectory data from the feature space, we established an abstract mathematical model for global strategy using the ProMPs method.
3. Neural networks were trained using the BC method to achieve assembly task strategy learning within the task space.

This paper is organized as follows: In Section 2, we provide an overview of related work. Section 3 outlines the methodology for establishing the velocity assembly skill model and the imitation learning approach guided by skill knowledge. Next, in Section 4, we conduct both simulation experiments and real robot experiments to validate the proposed method's performance and analyze the experimental results. Finally, Section 5 summarizes the entire paper.

## 2. Related work

The proposed method in this paper mainly involves modeling with probabilistic motion primitives and utilizing simplified geometric feature representation. This enables robotic assembly tasks based on behavior cloning methods to achieve improved learning outcomes. Relevant work in this area includes research on robot task representation and robot imitation learning. Recent research achievements in this field are as follows.

### 2.1. Representation methods for peg-in-hole assembly tasks

In the field of robot task learning, methods for task representation play a crucial role. Due to the varying representation requirements posed by different task learning methods, several types of task representation methods have emerged to date. These methods mainly fall into three categories: feature-based representation, perception-based representation, and neural network-based abstract representation.

Feature-based representation categorizes task states into discrete types. For instance, in Tsuruoka et al. (1997), contact states are classified into discrete categories such as single-point contact, two-point contact, three-point contact, surface contact and so on. A simplified state representation containing only three contact state categories was proposed in Huang et al. (2020) for medium-gap dual-arm peg-in-hole assembly tasks. The classification in Park et al. (2017) can be seen as a simplified version of the one presented in Tsuruoka et al. (1997) for situations with smaller gaps. While discrete representation methods effectively incorporate geometric features into skill learning, the limited number of categories often hinders detailed task skill modeling.

Continuous perception-based representation methods, as demonstrated in Huang et al. (2020), establish mappings between pose samples and the corresponding contact forces manually. They then use force sensing information to estimate correct poses, which represent contact states. Work like Park et al. (2017) introduces five-dimensional contact force information within the three-dimensional representation, resulting in an eight-dimensional continuous perceptual variable to represent the state of reinforcement learning agents. Perception-based information can be directly obtained from sensors and comprehensively represents task information, including poses and contact forces. However, continuous perception information can appear redundant and cumbersome during abstract skill analysis due to the abstract nature of skills.

Abstract information representation, as suggested in Ding et al. (2019), introduces a reinforcement learning-based pose estimator. This estimator calculates probabilistic weights for the six-dimensional pose space using visual and force information, representing the state for high-precision peg-in-hole assembly tasks in reinforcement learning. A more advanced method for constructing continuous perceptual abstract information is proposed in Lee et al. (2020) using Variational Autoencoders (VAE). They encode multimodal sensory information, including visual images, depth information, robot force and position sensing information, and contact force information, into an abstract code using a neural network variational model encoder. This approach achieves continuous abstract representation for peg-in-hole assembly tasks. These studies utilize neural networks to represent assembly tasks as concise and abstract state information.

In our study (Zang et al., 2023), we propose a continuous and streamlined representation method for peg-in-hole assembly states by analyzing the geometric features. This method reduces the dimensionality of reinforcement learning for peg-in-hole assembly, simplifying the learning process. In this paper, we will continue to use this abstract representation method to learn human assembly skills under geometric feature representation, aiming to acquire more global assembly skills from sparse human demonstrations.

### 2.2. Imitation learning methods for robot manipulation tasks

As mentioned earlier, imitation learning methods are primarily categorized into those based on mathematical models and those based on neural networks.

In mathematical analysis-based imitation learning methods, DMP are one of the earliest and most commonly used approaches. In Liu et al. (2020), the DMP imitation learning method was employed to extract the variations in trajectory characteristics as skill parameters, which were then used for reproducing demonstrated trajectories. Yang et al. (2019) utilized the DMP method for skill parameter extraction and operation replication based on human trajectories and stiffness information. However, DMP methods are limited to extracting data features and do not capture specific task features. Consequently, during the replication and generalization processes, they fail to retain the task characteristics inherent in demonstrated information, making it challenging to effectively generalize to similar tasks. Paraschos et al. (2018) introduced the ProMPs, a derivative of DMP, to extract probabilistic movement primitives of trajectories as skill parameters, facilitating the extraction of operation skill probability distribution parameters. Although ProMPs can retain probabilistic task-related features through probability calculations, the dimensionality redundancy in perception-based task representation compared to task features leads to the inefficient extraction of some task characteristics. Rozo et al. (2016) used the TP-GMM method to extract the Gaussian Mixture Model (GMM) of trajectories in the task coordinate system as skill parameters. Subsequently, they used Gaussian Mixture Regression (GMR) to replicate demonstrated operations from these skill parameters. While this method retains task-related features, its probability distribution model is still affected by the dimensionality redundancy in perception-based information representation.

With the rise of artificial intelligence technology, neural network-based imitation learning methods have gained widespread application in recent years. Li et al. (2023) employed the BC method to extract and replicate human driving skill parameters, enabling driving skill learning. Bhattacharyya et al. (2023) utilized GAIL to extract skill parameters from different driving styles and achieve replication of various driving styles. Additionally, Kim et al. (2020) introduced the Neural-Network-based Movement Primitive (NNMP) method, which models DMP using neural networks to retain task characteristics. However, the skill parameters obtained from neural network-based imitation learning methods are implicit (uninterpretable) neural network parameters ref8. These parameters not only fail to retain task characteristics but also exhibit limited generalization performance, making them unsuitable for extracting explicit, generic assembly skills.

In this paper, we will continue to learn assembly skills from human demonstration information using imitation learning methods, building upon the geometric feature space of peg-in-hole assembly. This approach aims to improve the performance of traditional imitation learning methods.

## 3. Method

In our previous work (Zang et al., 2023), we discovered that modeling task skills within a simplified geometric feature representation space allows for more comprehensive skill learning within the task space. In this paper, we will use traditional geometric-based imitation learning methods, ProMPs, and Behavioral Cloning (BC), to learn fundamental peg-in-hole assembly skills.

Unlike the previous work, this paper takes a different approach. Instead of directly specifying simple assembly skills, we will acquire skills from human demonstration information and engage in direct imitation learning. While this method involves human intervention, it aligns with practical applications where the same assembly task may require different skills under varying task requirements. Therefore, this approach holds promising prospects for learning specific assembly skills in particular application scenarios.

### 3.1. Peg-in-hole assembly ProMPs under geometric representation

In this paper, we will utilize a simplified geometric feature representation of the peg-in-hole assembly task as the task state. Firstly, for ease of calculation, regardless of whether the experiment fixes the peg or the hole component, we assume the peg component remains fixed, and the hole component is mobile when analyzing relative poses.

We denote the relative pose between the peg and the hole as Equation (1). And we have *R*_*ho*_ = [*n*_*x*_, *n*_*y*_, *n*_*z*_], $p_{ho}={\left[p_{x},p_{y},p_{z}\right]}^{T}$.


(1) $${}^{ax}T_{ho}=\left[\begin{array}{cc} R_{ho} & p_{ho}\\ 0 & 1 \end{array}\right]$$

During the calculation process, we map it to the geometric feature task space following the method described in Zang et al. (2023), and then get the representation information *Y* = {*x, z*, α, β, ϕ, θ} according to Equation (2).


(2) $$\begin{array}{ll} x & =\sqrt{p_{x}^{2}+p_{y}^{2}} \end{array}$$

(3) $$\begin{array}{ll} z & =p_{z} \end{array}$$

(4) $$\begin{array}{ll} \varphi & =\text{arctan}\left(p_{x}/p_{y}\right) \end{array}$$

(5) $$\begin{array}{ll} \theta & =\text{arctan}\left(n_{x}\left(3\right)/n_{y}\left(3\right)\right) \end{array}$$

(6) $$\begin{array}{ll} \alpha & =<n_{x}^{\prime },\left[-\text{sin}\varphi ,\text{cos}\varphi ,0\right]> \end{array}$$

(7) $$\begin{array}{ll} \beta & =<n_{z},\left[0,0,-1\right]> \end{array}$$

where $n_{x}^{\prime }$ can be denoted as Equation (8).


(8) $$\begin{array}{l} n_{x}^{\prime }=R_{ho}{\left[\begin{array}{ccc} \text{cos}\theta & -\text{sin}\theta & 0 \end{array}\right]}^{T} \end{array}$$

Through the aforementioned method, we can transform the obtained homogeneous transformation matrices into geometric feature representation information.

However, since the sensory information from human demonstrations is not always perfect, before extracting motion primitives from the sequence *Y*(*N*) of relative pose geometric representations, we first apply DTW to the trajectories. After DTW processing, we interpolate the geometric representation sequence as *Y*^*DTW*^(*N*′). In this context, the DTW distance function is defined as the Euclidean distance between the first four elements of the geometric representation sequence, denoted as $Y_{1:4}^{DTW}=\left\{x,z,\alpha ,\beta \right\}$. The distance between any two sequence points ^1^*P*^*DTW*^ and ^2^*P*^*DTW*^, and is expressed as Equation (9):


(9) $$\begin{array}{l} Dist=|{|}^{1}Y_{1:4}^{DTW}{-}^{2}Y_{1:4}^{DTW}|| \end{array}$$

This DTW processing allows us to handle imperfections in the human demonstration's sensory data, enabling us to obtain a more refined geometric representation sequence for further analysis and motion primitive extraction.

Then, for every dimension k of $Y_{1:4}^{DTW}$, we calculate the ProMP model, which are denoted as follows.


(10) $$\begin{array}{ll} y_{k}^{DTW}\left(t\right) & =\Phi_{k}w_{k}+\epsilon_{y_{k}^{DTW}} \end{array}$$

(11) $$\begin{array}{ll} P\left(\tau_{k}||w_{k}\right) & =\underset{t}{\prod }N\left(y_{k}^{DTW}\left(t\right)||\Phi_{k}w_{k},\Sigma_{y_{k}^{DTW}}\right) \end{array}$$

Where $\Phi_{k}\in {\mathbb{R}}^{n}$ represents the basis functions for the four-dimensional geometric representation variable of ProMPs, with n denoting the number of basis functions, and *w*_*k*_ as the corresponding weight vector. $\epsilon_{y_{k}^{DTW}}\sim N\left(0,\Sigma_{y_{k}^{DTW}}\right)$.

Next, we parameterize the weight parameters with Gaussian models θ_*k*_ = {*N*(μ_*w*_*k*__, Σ_*w*_*k*__)}. We then employ the Maximum Likelihood Estimation (MLE) method using the demonstrated trajectories ${}_{i}Y^{DTW}\left(N\right)$ to estimate the parameters of the Gaussian model for the weights. Here, *i* ∈ {1, 2, .., *N*_*dem*_} represents the index of the demonstrated trajectory. Subsequently, following the method described in Zang et al. (2023), we estimate the weights of the demonstrated trajectories through linear ridge regression. We use these weights to calculate the Gaussian model for the weight parameters.

### 3.2. Behavioral Cloning imitation learning method

In this paper, we will use the ProMPs model based on the geometric feature representation mentioned in Section 3.1 as the foundational model for generating operational knowledge samples under sparse demonstration data. We will then employ the BC method, using the knowledge samples to train a neural network for learning actions in different states. This is done to achieve the goal of data augmentation from demonstrations.

To facilitate practical applications, the input to the BC neural network consists of a seven-dimensional array *s*, composed of the three-dimensional relative position and the four-dimensional relative pose between the current peg and hole components. This is represented as follows:


(12) $$\begin{array}{l} s=\left\{p_{x},p_{y},p_{z},q_{x},q_{y},q_{z},q_{w}\right\} \end{array}$$

The output of the neural network corresponds to the executed actions and is structured as a six-dimensional array *a* as Equation (13). This array represents the change in the three-dimensional position and the change in the three-dimensional orientation angles of the end-effector.


(13) $$\begin{array}{l} a=\left\{\Delta p_{x},\Delta p_{y},\Delta p_{z},\Delta \theta_{x},\Delta \theta_{y},\Delta \theta_{z}\right\} \end{array}$$

Here, Δ*p* represent the changes in the end-effector's three-dimensional position, and Δθ represent the changes in its three-dimensional orientation angles. These output values provide the necessary information for executing actions in the context of the assembly task.

Because ProMPs can facilitate the learning of trajectory shapes, especially when waypoints are specified, it outperforms other imitation learning methods in terms of trajectory shape learning. In the case of neural network imitation learning methods based on state and action modeling, when a specific state *s* is determined, it is equivalent to specifying a waypoint on the trajectory. Consequently, ProMPs can be used to determine the knowledge action accordingly. However, one challenge that remains is how to determine the time point at which these waypoints occur.

In this paper, we will divide the trajectories aligned by DTW into several time stages. Then, we will use Gaussian models of each time stage to estimate which time stage the current state approximately corresponds to. This allows us to roughly determine the time point associated with the current state.

We suppose the geometric representation information corresponding to the trajectory state in a certain time stage is $Y^{DTW}\left(t_{s}\right)$, where *t*_*start*_ ≤ *t*_*s*_ ≤ *t*_*end*_. These constraints in Equation (14) are used to ensure that the time stages are appropriately divided and cover the entire trajectory duration.


(14) $$\{\begin{array}{l} \forall t_{s},\left\|Y_{1:4}^{DTW}(t_{s})-Y_{1:4}^{DTW}(t_{start})\right\|\leq l_{thre}\\ t_{s}-t_{s}\leq t_{thre} \end{array}$$

Under the constraints mentioned above, we select the longest time stage, which divides the trajectory into different time stages. Here, we assume that there are *N*_*t*_ time stages.

We extract samples from different time stages $y_{1:4}^{DTW}\left(t_{s}\right)$ of the demonstrated trajectory and compute gaussian models $N\left(\mu_{y_{1:4}^{DTW}},\Sigma_{y_{1:4}^{DTW}}\right)$ for these samples in each time stage. After obtaining the representation information $y_{1:4}^{DTW}\left(t\right)$ the current state corresponding to, we calculate the probability that the current sample point belongs to each time stage. The time stage with the highest probability is considered the current time stage. The specific time point can be chosen from within the current time stage, and in this paper, we directly select the midpoint of the time stage.

Once the time point is determined, we set waypoints in ProMPs and generate a target trajectory. However, we don't need to know all the trajectory information; we only need to use the trajectory point at a specific time within the next time stage as the target point. Then, we calculate the action *a* required to reach the target point, which serves as the knowledge information sample.

After obtaining the knowledge action sample, we use the BC method to imitate and learn the assembly skills from the demonstrated knowledge, thereby acquiring the peg-in-hole assembly skill.

## 4. Experiments

To validate the effectiveness of our proposed assembly skill imitation learning method, we conducted both simulation experiments and real robot experiments. Below are the details of the experimental setup and results.

### 4.1. Acquisition of demonstration information

**Figure 1:**
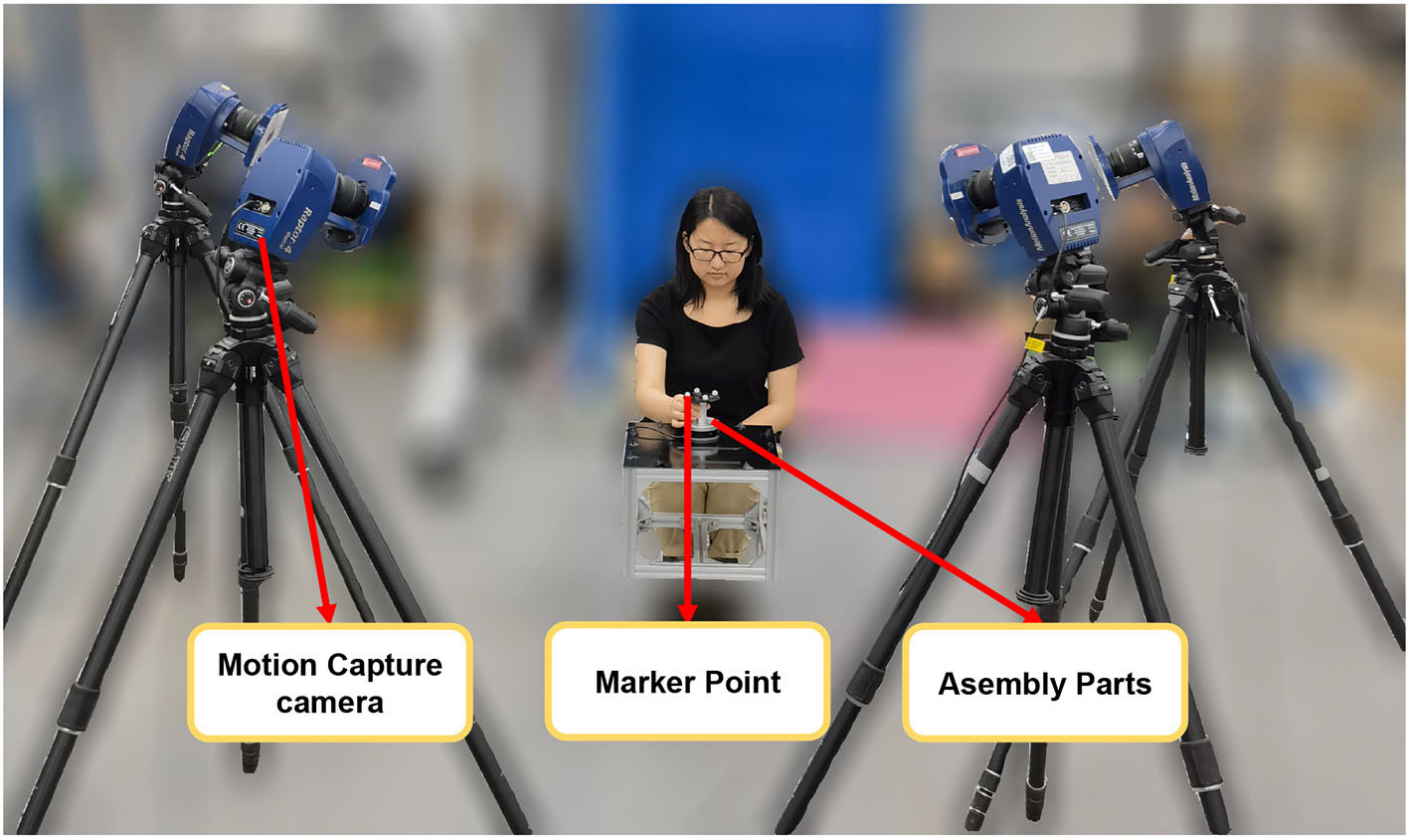
The picture of demonstration data acquisition equipments and the assembly parts.

**Figure 2:**
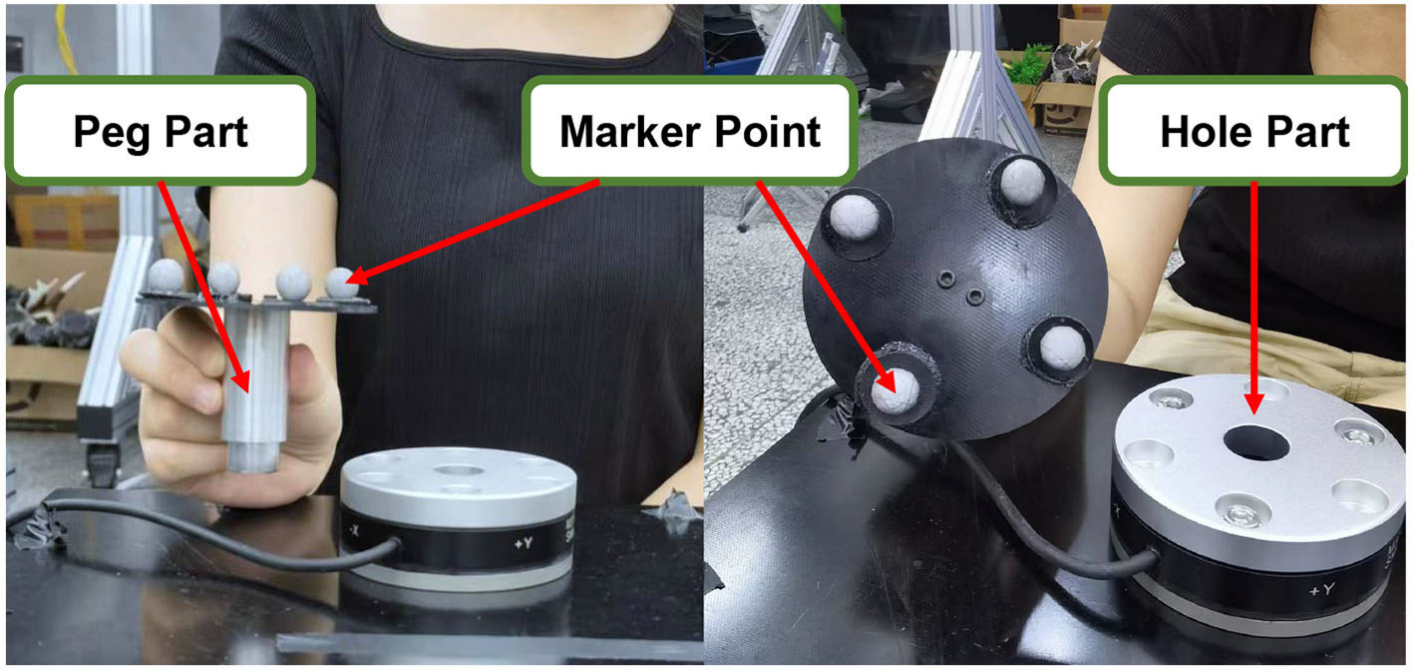
The picture of the assembly parts and the distribution of marker points.

To better record human demonstration information and minimize the impact of robot stalling and damping during the teaching process, we utilized motion capture equipment to capture the relative poses of the peg and hole during the human assembly process. The experimental setup is illustrated in Figure 1, while detailed schematics of the markers and the peg-in-hole components are shown in Figure 2.

**Figure 3:**
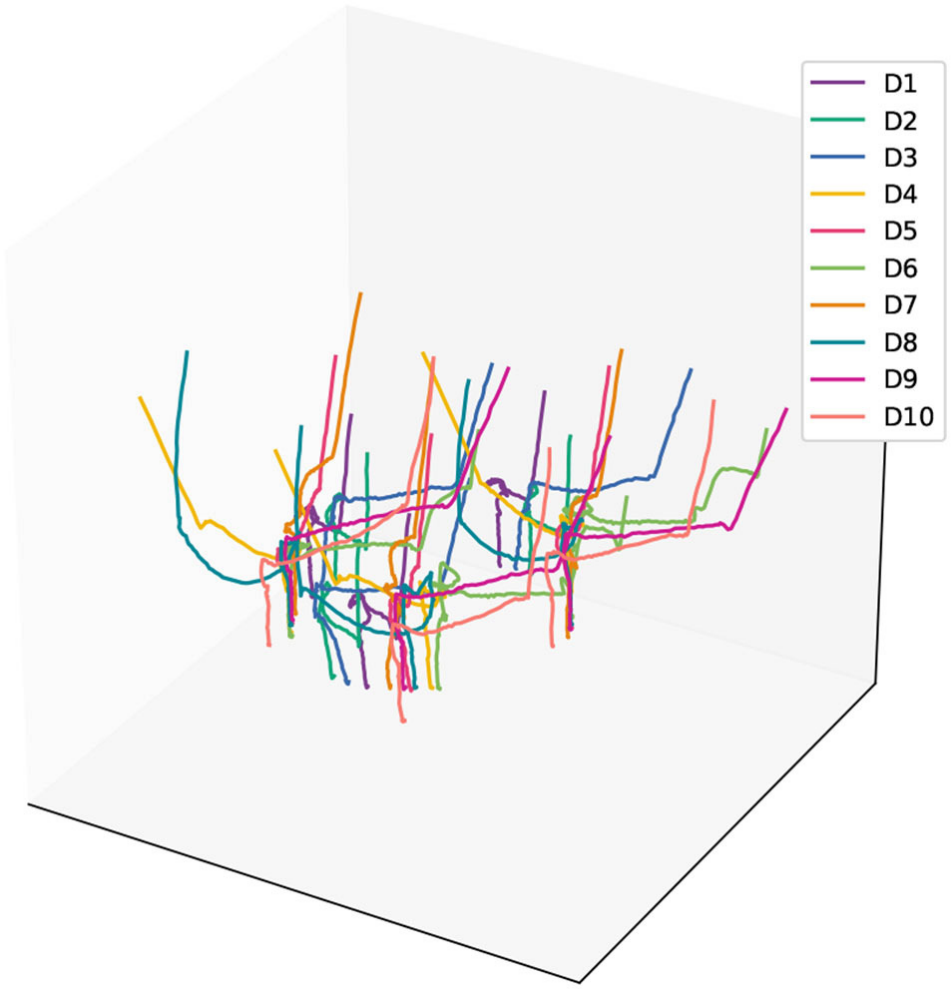
The recorded trajectories of the marker points during the human assembly demonstration.

During the data acquisition process for demonstration information, we manually held the peg component and executed the assembly strategy, which involved approaching the hole component along the axis of the hole and inserting it. We performed multiple assembly operations while capturing the position information of the markers. The trajectories of all the markers obtained from these operations are depicted in Figure 3.

**Figure 4:**
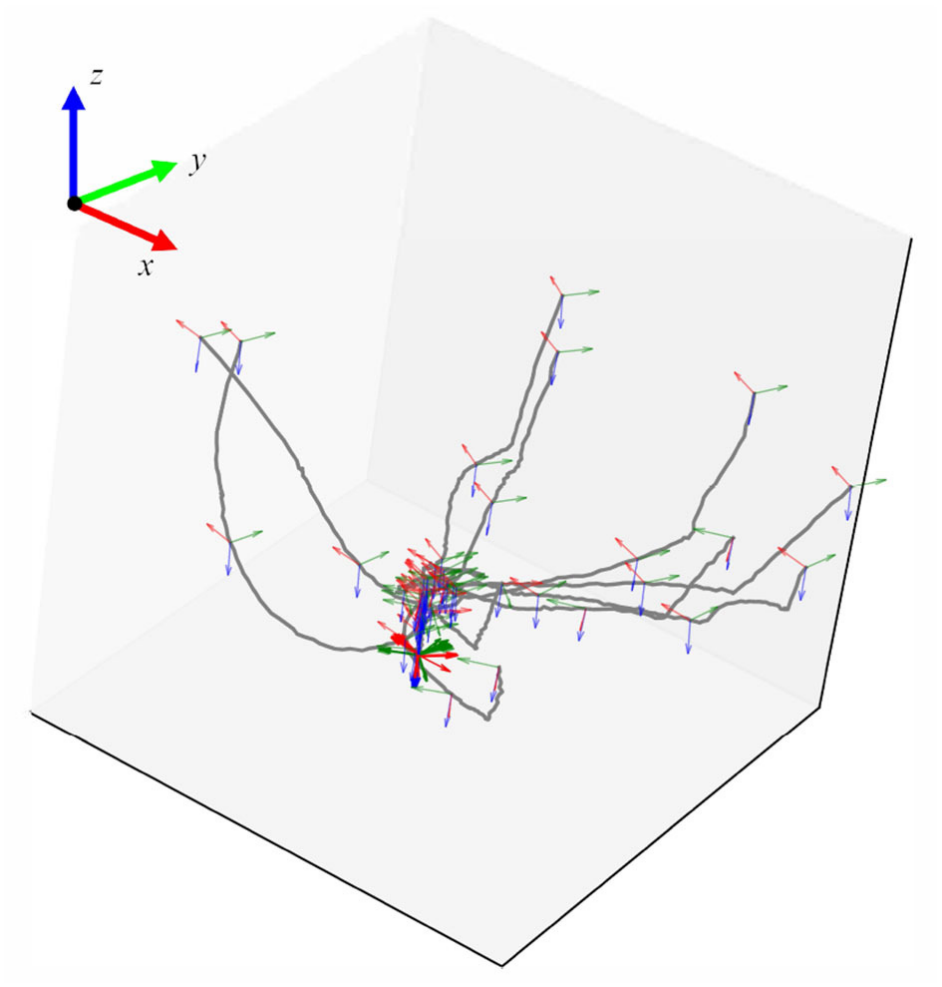
The processed trajectory data which denoted the pose of the peg part during the assembly demonstration.

By using the positions of four square-distributed markers, we calculated the homogeneous transformation matrix representing the pose of the peg component during the assembly process. Finally, from the obtained sensor data, we extracted the raw trajectory information of the demonstrated operation. After processing, the trajectory plot is shown in Figure 4.

### 4.2. Verify experiment of assembly skills imitaiton learning

**Figure 5:**
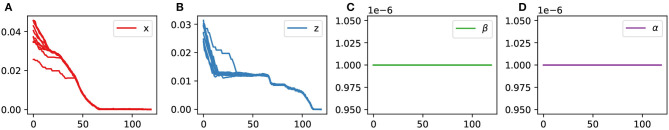
The aligned trajectories (processed by DTW) in geometric representation space. **(A–D)** Are the demonstration trajectories for different dimensions under geometric representation.

After obtaining the raw trajectory information, we mapped the trajectories into the geometric representation space and applied DTW for alignment. The aligned trajectories in the geometric representation space are depicted in Figure 5. In this figure, it can be observed that the demonstrated operation trajectories align effectively within the geometric feature representation space, forming a concentrated set of knowledge trajectories.

**Figure 6:**
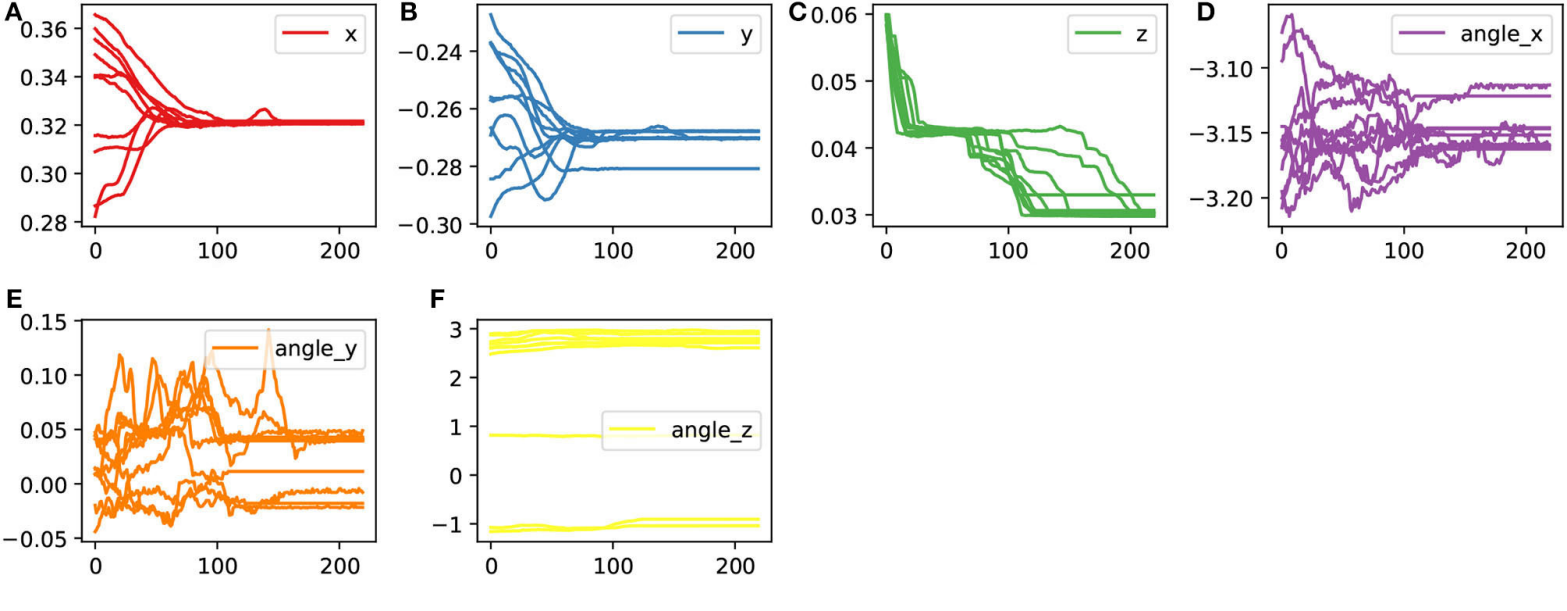
The aligned trajectories (processed by DTW) in the cartesian representation space. **(A–F)** Are the demonstration trajectories in different cartesian dimensions.

As a comparison, we also aligned the trajectories in cartesian representation space, as shown in Figure 6.

As shown in the figure, under the geometric feature representation, the demonstrated trajectories are more easily aligned, resulting in a concentrated set of assembly skills.

**Figure 7:**
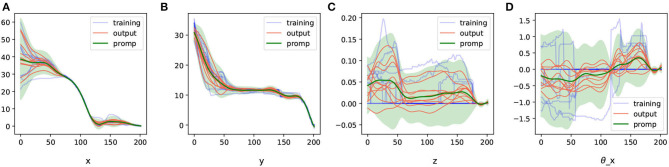
The ProMPs model in geometric representation space extracted from the aligned trajectories and the trajectories generated from them. **(A–D)** Are the ProMPs model for different dimensions under geometric representation.

We extracted ProMPs from the trajectory representation information processed with DTW and generated some random trajectories based on the model, as shown in Figure 7.

**Figure 8:**
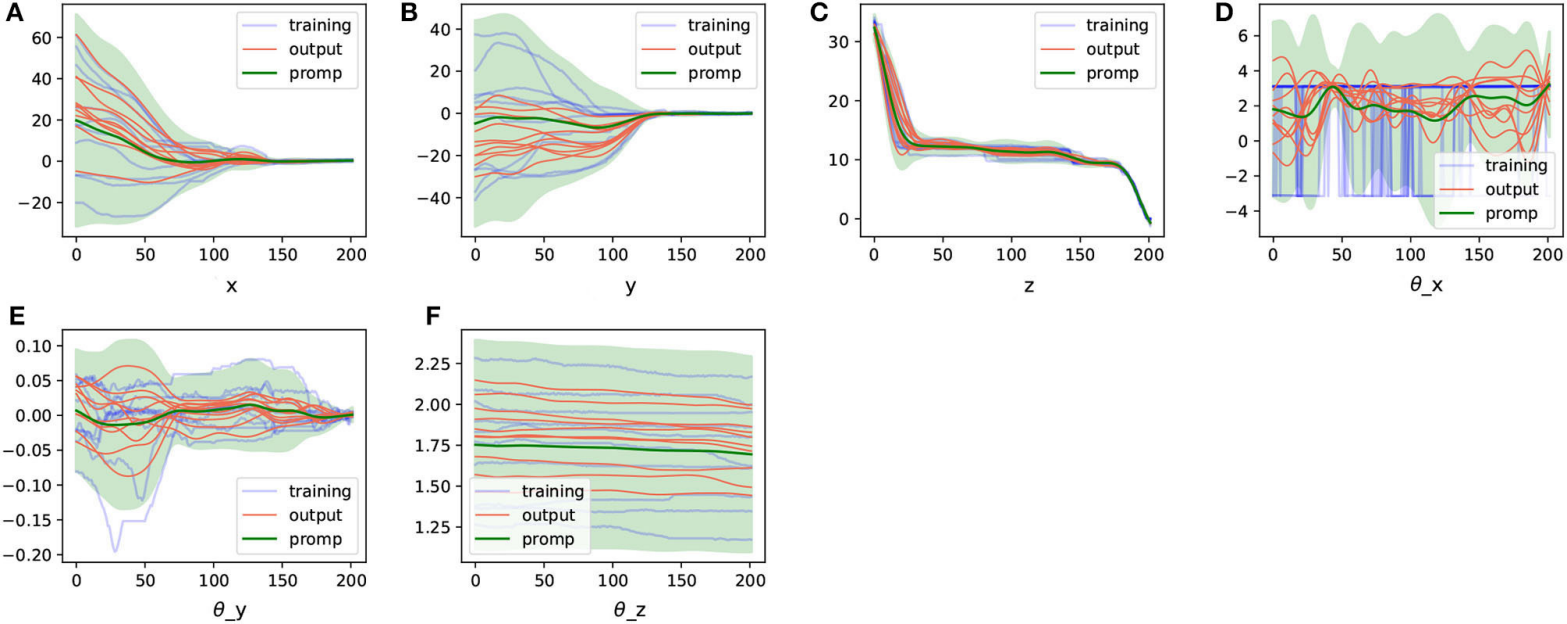
The ProMPs model in cartesian representation space extracted from the aligned trajectories and the trajectories generated from them. **(A–F)** Are the ProMPs model for different cartesian dimensions.

In comparison, we directly utilized the trajectories obtained in cartesian space representation to extract ProMPs and generated some random trajectories based on this model, as shown in Figure 8.

After obtaining the ProMPs model, we sampled several points in different task space representations. We used the current sampling point as a through point, generated task trajectories through this sampling point using the ProMPs model, and then took the target pose from the next time stage as the target, resulting in action sampling. We used this data to train a Behavioral Cloning neural network model. This way, we conducted two sets of imitation learning experiments in both the geometric representation task space and the Cartesian task space.

Additionally, we set up three sets of control experiments. The first one training a neural network for BC using only action samples from demonstrated trajectories. The second one used ProMPs under geometric representation to generate trajectories. The last one used ProMPs under cartesian representation to generate trajectories. In the end, we had the following five groups of experiments:

1. EXP_1: ProMPs model in task representation space + BC imitation learning.
2. EXP_2: ProMPs model in Cartesian representation space + BC imitation learning.
3. EXP_3: Naive BC imitation learning.
4. EXP_4: ProMPs model in geometric representation space.
5. EXP_5: ProMPs model in Cartesian representation space.

We trained the neural network in an environment based on Ubuntu and PyTorch. The neural network had six layers, with 100 neurons in each layer. The input to the neural network consisted of the current relative pose of the components, and the output was a six-dimensional action.

**Figure 9:**
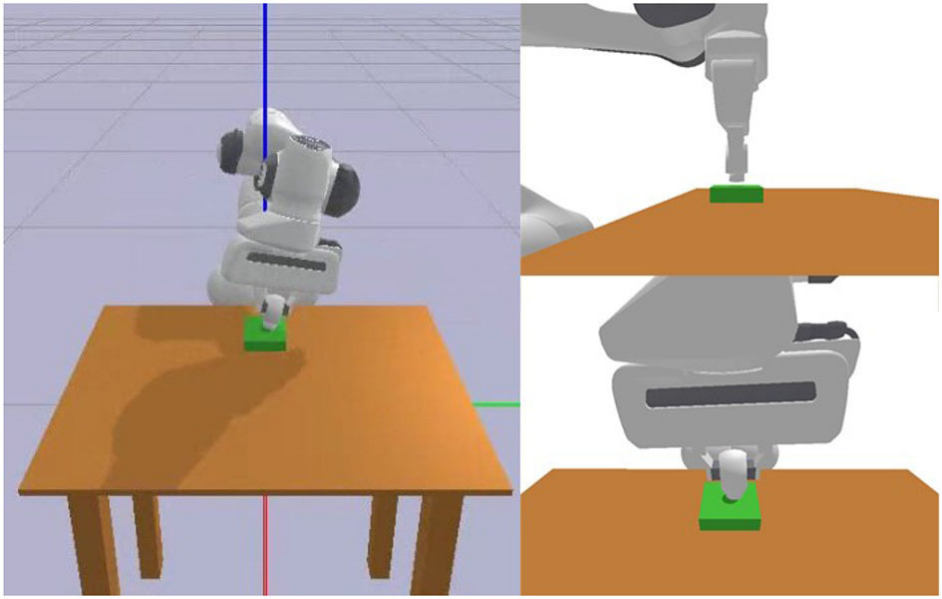
The picture of the simulation platform.

We conducted simulation experiments in the Pybullet environment, as shown in Figure 9. The experimental subject was the Franka Panda robot. We calculated the target pose based on the current action's position and angle increment and then calculated the robot's joint angle changes based on the target pose for position control. We used the Trac Ik method to calculate the robot's inverse kinematics.

**Figure 10:**
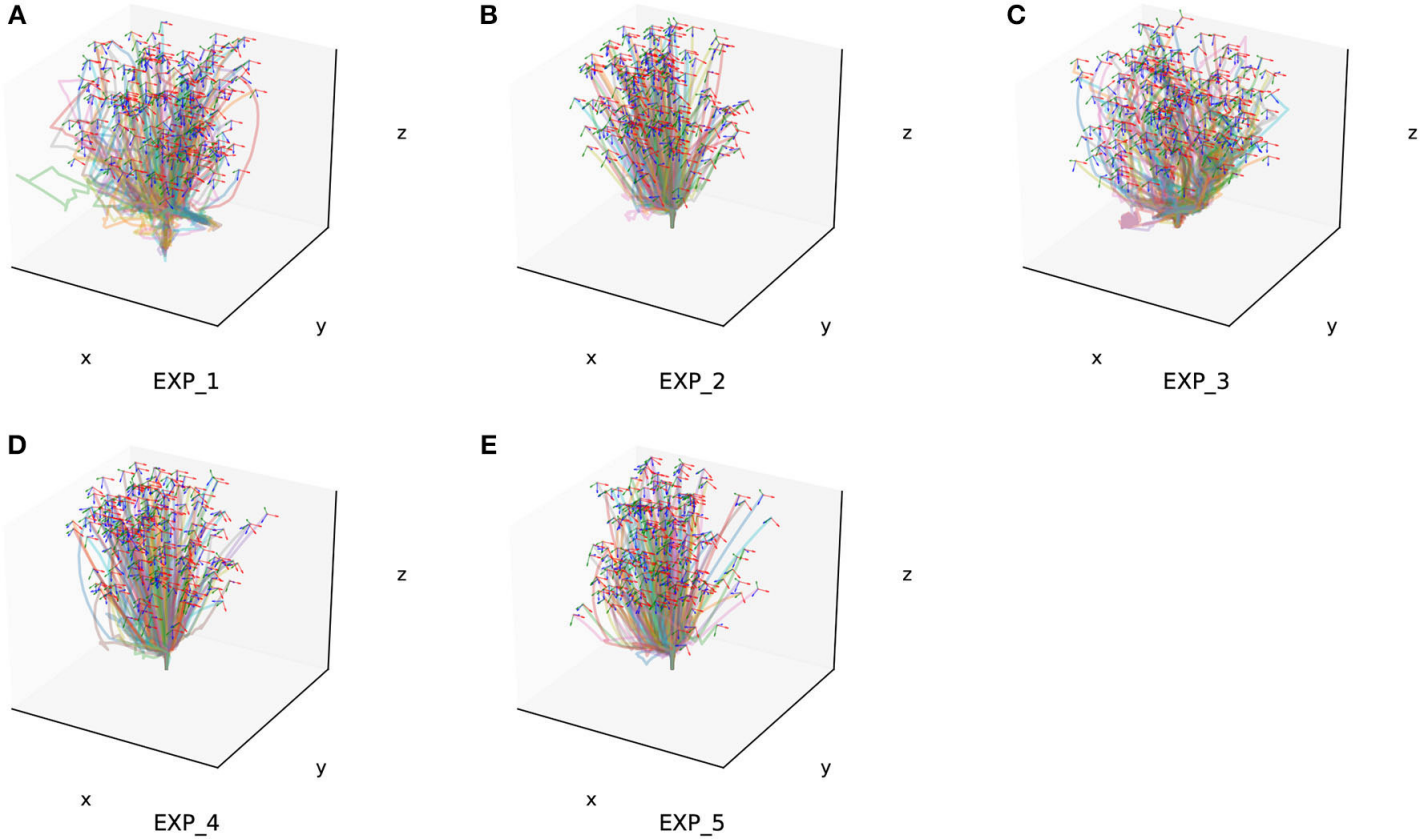
Two hundred of the success trajectories of each simulation experiment. **(A–E)** Is the verify experiment result for EXP_1–EXP_5.

The assembly components used have a diameter of 20 mm and consist of pegs and holes with a matching length of 12 mm. The gap between the pegs and holes is <0.5 mm. We randomly selected 500 sets of random state data within the task space and conducted assembly experiments for each of the four configurations. We choose 200 successful generated trajectories of each experiment randomly and show them in Figure 10.

**Figure 11:**
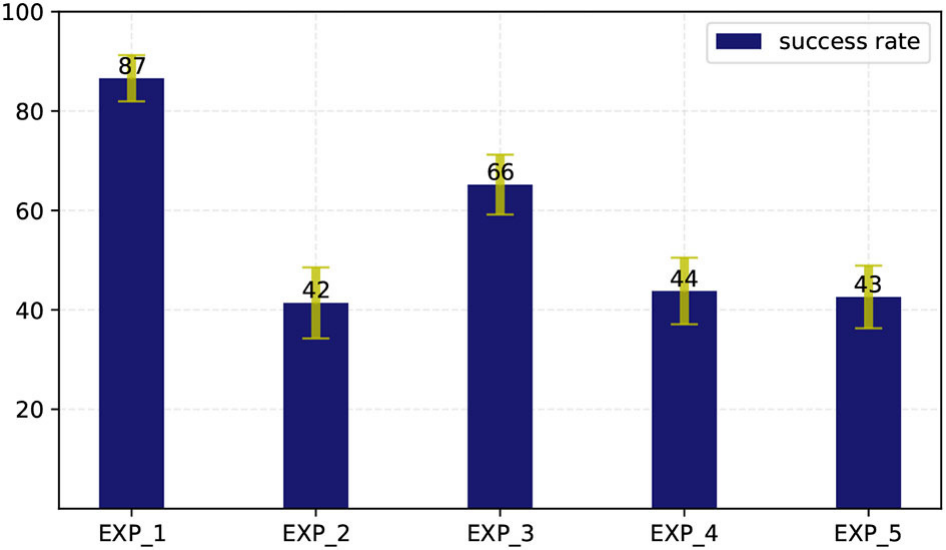
The success rate of each experiment as well as the variance of it.

The success rate of each experiment as well as the variance of it are shown in Figure 11.

**Figure 12:**
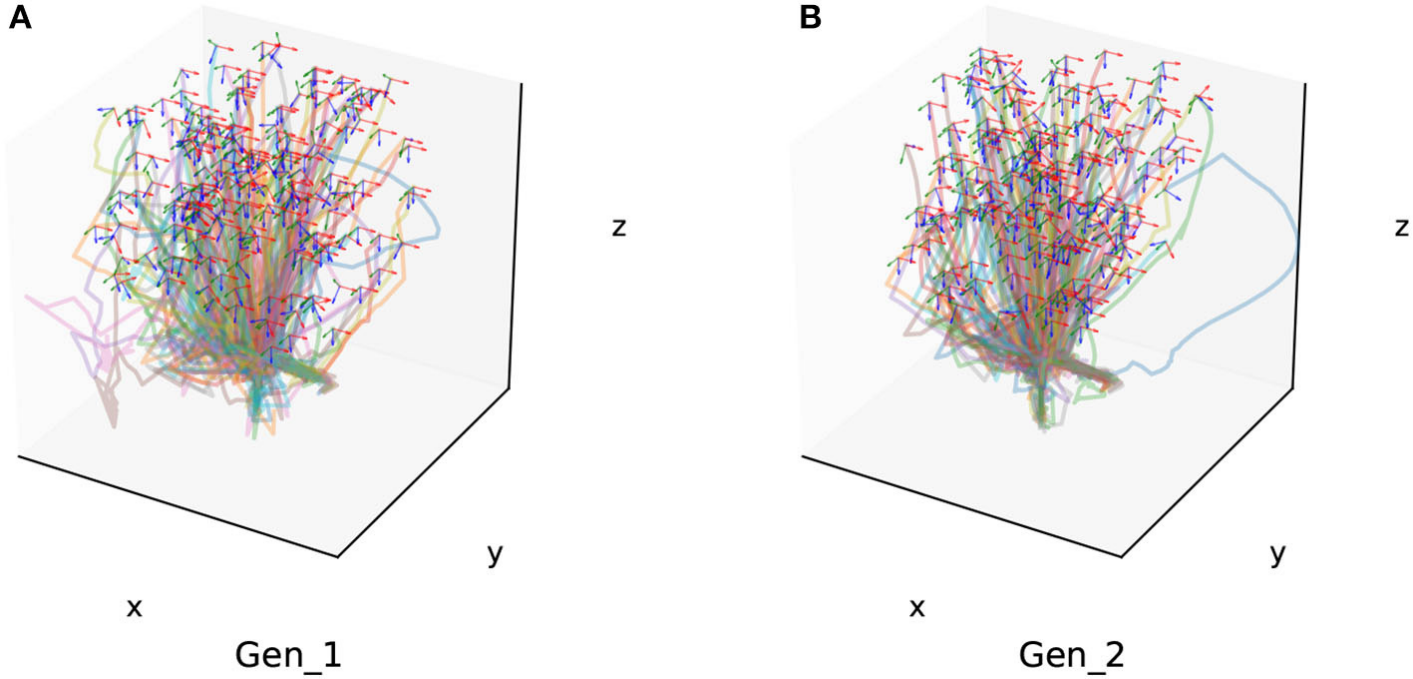
Two hundred of the success trajectories of each generalization experiment. **(A)** Is the generalization experiment result of peg-in-hole assembly task with 10 mm radius. **(B)** Is the result of peg-in-hole assembly task with 15 mm radius.

Additionally, we conducted generalization experiments for assembly components of different sizes as part of Experiment 1. This included assembly sizes with diameters of 10 mm (Gen_1) and 15 mm (Gen_2). Two hundred success trajectories are also chosen randomly from the generalization experiment result for each grop, which are shown in Figure 12.

**Figure 13:**
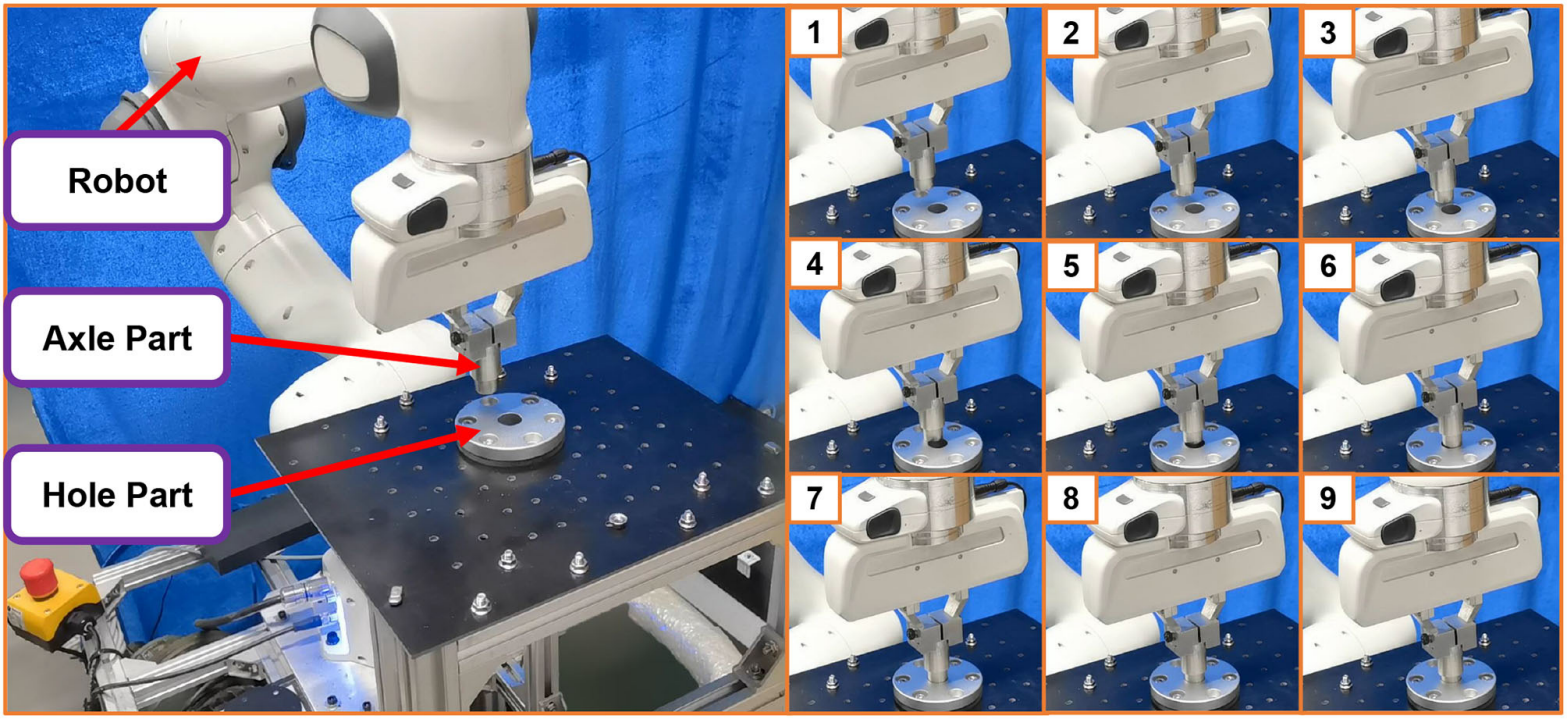
The picture of the real world experiment platform and the snapshots.

Finally, we performed real-world robot generalization experiments using the assembly actions learned from Experiment 1. The experimental setup and results in the form of time-series graphs are illustrated in Figure 13. Notalby, in real world experiment, we use the impandance control instead of position control because of the collision of the peg-in-hole task. We demonstrated that our proposed imitation learning method for robot peg-in-hole assembly tasks, based on geometric representations and ProMPs, can effectively learn assembly strategies and achieve higher performance compared to traditional methods.

### 4.3. Discussion

For the simulation experiments, we found that the imitation learning method based solely on the ProMPs approach had certain limitations in global task learning, whether in the task representation or Cartesian representation. This suggests that the ProMPs model itself has limitations in trajectory generalization. In other words, when the selected state as a passing point significantly deviates from the original ProMPs model's trajectory, the generalized trajectories generated by the ProMPs model may struggle to meet the requirements of tasks with rich contacts. Additionally, the similar success rates obtained in Experiment 4 and Experiment 5 indicate that the ProMPs model's performance does not differ significantly in different task representations. We believe this is because both experiments are based on learning the same demonstrated trajectories, so the learned trajectory shapes are generally similar regardless of the representation.

However, this does not imply that the ProMPs model cannot be an effective method for obtaining generalized trajectories. On the contrary, the higher success rate achieved in Experiment 1 using the ProMPs model with geometric feature representation for BC imitation learning suggests that the ProMPs model can yield high success rates when used in combination with specific task representations. Nonetheless, we observe that even when both experiments use the ProMPs model for BC imitation learning, the success rate in the Cartesian space representation is not ideal. We attribute this to two factors. First, the ProMPs model in Cartesian space has higher variance, and the knowledge is not as concentrated. Additionally, the knowledge in the geometric feature representation is more concise, making it easier to obtain a uniform global skill, which is beneficial for neural network learning. Therefore, we conclude that geometric feature representation plays a crucial role in neural network-based task learning.

Finally, the surprising results obtained in Experiment 3 with the naive BC imitation learning agent, although slightly inferior to Experiment 1, can be attributed to the simplicity of the provided skill. Moreover, the demonstration data effectively covered the task space, allowing the neural network's generalization capabilities to be effectively utilized, leading to better results.

## 5. Conclusion

In this paper, we have introduced a robot assembly task imitation learning method based on ProMPs under a task-specific representation. This method involves the use of probabilistic movement primitives in the geometric feature representation and BC imitation learning based on the ProMPs model. We have conducted comparative experiments to demonstrate the effectiveness of our proposed approach and provided generalization experiments. Finally, we have analyzed the experimental results and offered our insights into the factors contributing to the outcomes in different experiments.

While the method presented in this paper has shown promising results in the current task setting, it still has some limitations in practical applications. Therefore, in future work, we aim to integrate the skill modeling capabilities of imitation learning methods with the generalization capabilities of neural networks. We plan to investigate assembly task learning methods for different task settings to address these limitations and enhance the applicability of the approach.

## Data availability statement

The raw data supporting the conclusions of this article will be made available by the authors, without undue reservation.

## Ethics statement

Written informed consent was obtained from the individual(s) for the publication of any identifiable images or data included in this article.

## Author contributions

YZ: Conceptualization, Data curation, Formal analysis, Investigation, Methodology, Software, Validation, Visualization, Writing—original draft. PW: Conceptualization, Investigation, Project administration, Supervision, Writing—review & editing. FZ: Conceptualization, Data curation, Formal analysis, Funding acquisition, Investigation, Methodology, Project administration, Resources, Supervision, Writing—review & editing. WG: Conceptualization, Data curation, Investigation, Methodology, Project administration, Supervision, Writing—review & editing. CZ: Funding acquisition, Resources, Supervision, Writing—review & editing. LS: Conceptualization, Funding acquisition, Investigation, Methodology, Project administration, Resources, Supervision, Writing—review & editing.
